# Modeling Antibody Kinetics Post‐mRNA Booster Vaccination and Protection Durations Against SARS‐CoV‐2 Infection

**DOI:** 10.1002/jmv.70521

**Published:** 2025-08-06

**Authors:** Luis J. Ponce, Yuqian Wang, Ananya Singh, Hoong Kai Chua, Marc Chen, Pei Xiang Hor, Chiew Yee Loh, Xuan Ying Poh, Suma Rao, Po Ying Chia, Sean W. X. Ong, Tau Hong Lee, Ray J. H. Lin, Clarissa Lim, Jefanie Teo, Yun Shan Goh, Keisuke Ejima, on behalf of the NCID Study Group, on behalf of the COVID‐19 Cohort Study Group

**Affiliations:** ^1^ Lee Kong Chian School of Medicine Nanyang Technological University Singapore Singapore; ^2^ School of Biological Sciences Nanyang Technological University Singapore Singapore; ^3^ A*STAR Infectious Diseases Labs (A*STAR ID Labs), Agency for Science, Technology, and Research (A*STAR) Singapore Singapore; ^4^ National Center for Infectious Diseases (NCID) Singapore Singapore; ^5^ Department of Infectious Diseases Tan Tock Seng Hospital Singapore Singapore; ^6^ National Healthcare Group Singapore Singapore

**Keywords:** COVID‐19 breakthrough infections, COVID‐19 vaccines, immunoglobulin A, immunoglobulin G, SARS‐CoV‐2, survival analysis

## Abstract

Understanding the dynamics of SARS‐CoV‐2 antibody levels post‐booster vaccination is important to inform their durations of protection. Longitudinal antibody data was collected on the day of booster vaccination, as well as 28, 180, and 360 days after. Using nonlinear mixed effects models, we mapped the kinetics of binding IgA and IgG against wild‐type (WT) and Omicron BA.1 spike proteins. Furthermore, we analyzed the association between antibody levels and risk of SARS‐CoV‐2 vaccine breakthrough infection through survival analyzes, and predicted durations of protection against infection. We found that the antibody response waned more rapidly following the Pfizer/BioNTech BNT162b2 booster compared to the Moderna mRNA‐1273 booster. However, individuals boosted with the Pfizer vaccine exhibited a steeper rebound in antibody levels after infection. Faster postinfection antibody growth rates were observed in the elderly, females, and those with late infections. High antibody levels for WT IgG and BA.1 IgA at day 28 post‐booster were associated with reduced infection risk; hazard ratios were 0.47 (95% CI [0.22, 0.98]) and 0.36 (95% CI [0.17, 0.78]), respectively, compared to low levels. Time‐varying antibody levels showed better survival model fits. At medium COVID‐19 case incidence (621 cases per million per day), a binding BA.1 IgA response of at least 20% is needed to sustain 80% protection against infection over 155 days post‐booster. Our estimates of protection durations against SARS‐CoV‐2 infection post‐booster vaccination may help inform the ideal frequency of boosters.

## 1 Introduction

Severe acute respiratory syndrome coronavirus 2 (SARS‐CoV‐2), the virus responsible for coronavirus disease 2019 (COVID‐19), is a highly infectious respiratory virus that primarily spreads through air droplets or contact surfaces [1]. While most infected people recover without hospitalization, the disease can be particularly severe or fatal in older adults, unvaccinated individuals, and those with underlying health conditions [2, 3]. Since the COVID‐19 pandemic began in early 2020, in addition to contact tracing and isolation measures, the global burden of severe COVID‐19 was significantly mitigated by widespread population immunity from mass vaccinations [4]. By 31 December 2023, 67% of the global population had completed a primary series of COVID‐19 vaccination against the ancestral Wuhan wild‐type (WT) strain [5]. However, because of the emergence of new SARS‐CoV‐2 variants (such as Omicron BA.1), coupled with the inevitable waning of immune responses, vaccine effectiveness decreases over time [6]. To counteract such waning, the administration of additional vaccination doses (boosters) to protect against specific circulating viral strains has been useful.

Exposure to antigens, either via vaccination or infection, results in the induction of host immune responses, providing protection against infection and severe COVID‐19 [7]. Several studies have identified the contribution of the different arms of the immune system in the protection against SARS‐CoV‐2 infection [7, 8, 9, 10, 11, 12, 13]. The combination of CD4^+^/CD8^+^ T helper cells and vaccine/infection‐induced binding and neutralizing antibodies has been postulated as the main contributors of protection against infection [9]. Many studies have also explored the trajectories of antibody responses after vaccination or infection. Their findings generally agree on the time it takes to reach peak antibody levels, but they highlight significant differences depending on the time since the last vaccination, with greater protection after infection compared to vaccination [8, 14, 15, 16]. Additionally, they show that a third immunizing dose offers greater durability of protection compared to a second dose, and they provide estimates for how long protection lasts after just two doses [17, 18].

Antibody kinetics is essential for understanding its role in protection against infection. Strong evidence for antibodies providing protection against infection has been well‐established, yet the dynamic nature of antibody levels over time solicits further analysis to inform potential windows of protection. As antibody levels and associated vaccine effectiveness wane over time, booster vaccination is essential [19]. However, to inform the appropriate timing of booster vaccinations, we must understand the association between the time‐varying antibody response and infection risk. Although many cohort studies have measured antibody levels over time [7, 19], evaluating the association between antibody levels and infection risk is challenging, in part due to antibody levels constantly changing over time without realistically being able to repeatedly sample and measure them, especially at the timing of infection. Additionally, studies that model the antibody kinetics when Omicron is the dominant circulating variant are rare. As of May 2025, JN.1 and its descendants KP.2, KP.3, XEC, and LP.8.1—sublineages of the Omicron family—remain among the dominant circulating variants, and it is still unclear when the current Omicron‐driven transmission pattern will subside. As new boosters are being updated based on the latest circulating Omicron subvariant, this highlights the importance of robust models for examining antibody responses and infection risk against Omicron and its subvariants.

Previous studies have evaluated antibodies as correlates of protection against infection, comprehensively distinguishing between antibody isotypes and the viral variants they target [9, 11, 12, 19]. These investigations consistently indicate that higher levels of anti‐spike (S) IgG and IgA against the Wuhan WT, Delta, and Omicron variants are associated with a reduced risk of breakthrough infection. For instance, elevated IgG levels against the Omicron S protein have shown a hazard ratio (HR) of 0.06 (95% CI [0.01, 0.26]) per log_10_‐transformed median fluorescence intensity [11]. Another study observed that an additional booster vaccination reduced the risk of symptomatic infection by 37% (95% CI [15%, 54%]) [12]. Existing studies have primarily relied on booster response antibody levels (i.e., antibody levels 2‐3 weeks after vaccination) to assess infection risk; however, incorporating time‐varying antibody levels following the booster response could offer deeper insights. While booster response levels as predictors in survival analyzes may provide a snapshot at a specific timepoint, transforming antibody levels into time‐varying predictors can capture the dynamic immune response, revealing fluctuations that may better correlate with changes in infection risk. This approach could uncover critical windows of vulnerability or enhanced protection, as well as the timing or time interval of booster vaccination, informing finer vaccination strategies.

Mathematical models have been used to estimate the temporal dynamics of biomarkers, and they reflect the biological mechanisms that occur within individuals after vaccination or infection. For example, they can capture well the processes of virus replication and elimination that occurs due to the immunological response, or the changes in antibody levels after receiving a vaccine [20, 21, 22, 23, 24, 25, 26, 27, 28, 29, 30, 31, 32, 33, 34]. In this study, we develop a mathematical model that describes the temporal changes in antibody levels, specifically the increase in antibody levels due to booster vaccination and infection as well as the subsequent antibody waning over time. Additionally, we examine the association between the booster response and time‐varying antibody levels and infection risk through survival analyzes.

To justify our model structure, we leveraged the observed pattern that antibody levels rise sharply following booster vaccination or infection before declining over time. This biphasic pattern—initial boosting followed by waning—is well‐established and provides a biological basis for including both booster‐ and infection‐induced antibody responses, along with their respective decay rates, in our model. By fitting this structure to longitudinal antibody data, we were able to estimate individual‐level antibody trajectories that reflect both immune stimulation and waning phases. These personalized trajectories were then incorporated as time‐varying covariates in survival models to evaluate how fluctuating antibody levels influence the risk of infection. This integrated framework enables us to link immunological dynamics with real‐world protection, offering insights into how long immunity lasts and when individuals may become susceptible to breakthrough infection.

## 2 Methods

### 2.1 Sex as a Biological Variable

Our study examined both male and female individuals, and sex was considered as a potential covariate in the analyzes.

### 2.2 Study Participants and Sample Collection

**Figure 1:**
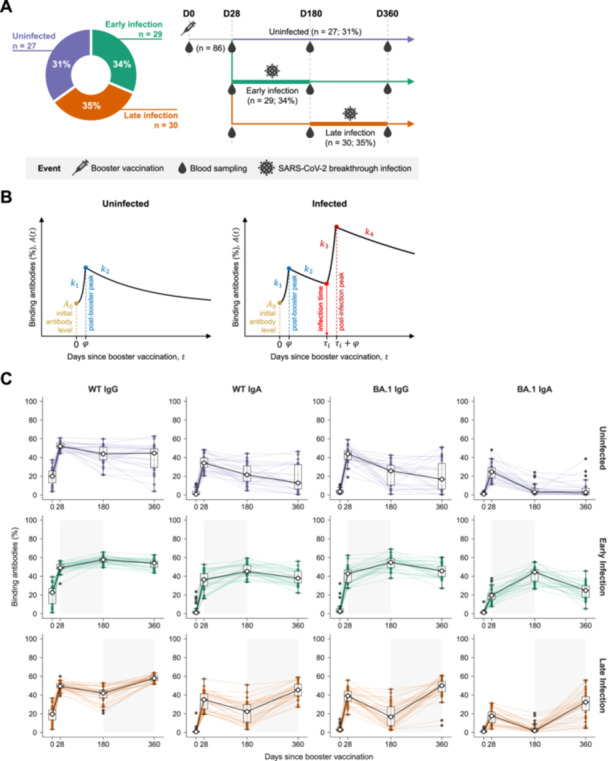
Overview of the study design and methodology. (A) Schematic timeline of booster vaccination, blood sample collection, and SARS‐CoV‐2 infection events. The x‐axis represents time in days following the booster vaccination (Day 0). Blood samples were collected at four time points: day of booster shot, and 28, 180, and 360 days post booster vaccination for antibody measurements (referred to as D0, D28, D180, and D360, respectively). Participants were classified into three groups based on the timing of SARS‐CoV‐2 infection during the follow‐up period: (1) early infection (infected between D28 and D180 post‐booster vaccination; *n* = 29, 34%), (2) late infection (infected between D180 and D360 post‐booster vaccination; *n* = 30, 35%), and (3) uninfected (remained uninfected throughout follow‐up period post‐booster vaccination; *n* = 27, 31%). No infections were observed between D0 and D28. (B) Schematic of the antibody dynamics with associated key model parameters. Antibody levels increase at an exponential rate $k_{1}$ from the initial level $A_{0}$ and peak at φ days after booster vaccination, after which they decrease at an exponential rate $k_{2}$ until the end of the follow‐up period (for uninfected cases) or until infection at $\tau_{i}$ (for both early and late infections). For an infected individual, antibody levels will increase exponentially with a growth rate of $k_{3}$ before reaching a second higher peak at ($\tau_{i}+\varphi$) days, and eventually wane at a decay rate of $k_{4}$ until the end of the follow‐up period. (C) Observed antibody trends of IgG and IgA for the wild‐type (WT) and Omicron BA.1 variant, stratified by the timing of infection as described in (A). Trajectories are depicted as colored lines connecting the values from each sample timepoint for each participant, with boxplots depicting the median (50th percentile; solid line) and interquartile ranges (25th and 75th percentiles; boxes). The “typical” kinetics for each group are depicted by the black lines connecting the medians. Outlier points beyond 1.5 times the interquartile range (shown as whiskers) are plotted as black dots. The time interval where an infection event occurs for each group is shaded in gray.

**Figure 2:**
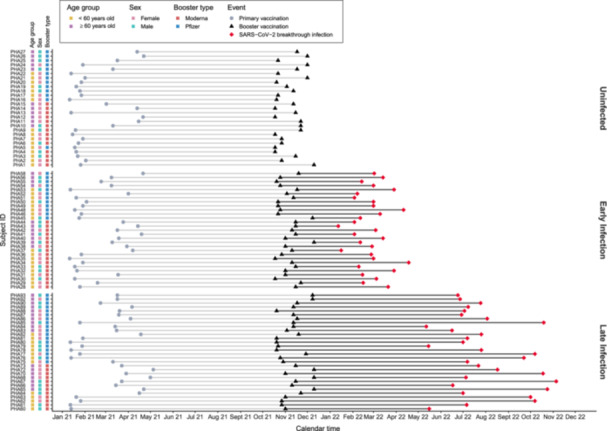
Schematic representation of the study timeline and individual participant characteristics. A summary of the analyzed longitudinal cohort data set is shown. All participants received their primary two‐dose mRNA vaccination (Pfizer/BioNTech BNT162b2) from January to May 2021 (indicated as gray circles), followed by their booster vaccination (Pfizer/BioNTech BNT162b2 or Moderna mRNA‐1273) at least six months later between October to December 2021 (indicated as black triangles). Participants were closely monitored from January to December 2022 to determine their SARS‐CoV‐2 infection status, which was stratified into one of three groups: (1) early infection (within 180 days of follow‐up), (2) late infection (between 180 and 360 days of follow‐up), or (3) uninfected (no infection within 360 days of follow‐up). Blood sample collection occurs from the day of booster vaccination onwards and infection events are indicated as red diamonds. The categories of each participant's age, sex, and type of booster vaccination received are also shown on the left.

A cohort of 93 adult individuals in Singapore was recruited as part of the PRIBIVAC study, a randomized controlled subject‐blinded trial to evaluate the immunogenicity and safety of heterologous booster vaccinations compared to homologous booster vaccinations (where the booter type was randomly assigned) [35]. This study has received ethical approval from the Institutional Review Board: National Healthcare Group Domain Specific Review Board (NHG DSRB, study reference 2021/00821). All participants had completed their primary two‐dose COVID‐19 mRNA vaccination series (Pfizer/BioNTech BNT162b2), and all were infection‐naive at the beginning of the study period. Each participant received their booster (third dose, WT) vaccination (either Pfizer/BioNTech BNT162b2 (30 μg) or Moderna mRNA‐1273 vaccine [50 μg]) between 18 October and 10 December 2021—six to twelve months after their primary vaccination. Blood samples were collected on the day of booster (before vaccine administration), as well as 28 days, 180 days, and 360 days thereafter. We refer to these sampling timepoints as D0, D28, D180, and D360, respectively (Figure 1A). A schematic overview of the study timeline and patient characteristics is provided in Figure 2.

During the follow‐up period, infection status was monitored through self‐reporting of a positive polymerase chain reaction (PCR) test or antigen rapid test (ART) and confirmed by the presence of antibodies against the nucleocapsid (N) protein (via Elecsys anti‐N assays performed at all blood collection time points). We further stratified participants into three main groups based on their infection history during the follow‐up period: (1) an “early infection” group for those infected between D28 and D180 post‐booster vaccination, (2) a “late infection” group for infections that occurred between D180 and D360 post‐booster vaccination, and (3) an “uninfected” group for those who remained uninfected over the entire follow‐up period post‐booster vaccination (Figure 1A).

For all subsequent analyzes, we excluded four participants with an unknown SARS‐CoV‐2 infection date, two participants with incomplete antibody data at D28, and one participant who was infected 1 year after receiving the booster dose (i.e., past the follow‐up period), thus the final number of participants analyzed is 86. Note that all participants received mRNA vaccines, and no participant received additional boosters beyond the third dose during the follow‐up period.

### 2.3 Detection of Anti‐S Antibody Response

Anti‐S antibody response was measured using the S protein flow‐based (SFB) assay as previously described [36, 37]. HEK293T cells expressing full‐length S protein of either the ancestral WT Wuhan or Omicron BA.1 were seeded at 1.5 × 10⁵ cells per well in 96‐well V‐bottom plates. Cells were incubated with human plasma samples—routinely diluted at 1:100 in 10% FBS—for 30 min at 4°C, followed by a secondary 30‐min incubation at 4°C with a double stain comprising Alexa Fluor 647‐conjugated anti‐human IgG or IgA (1:500 dilution) and propidium iodide (PI; 1:2500 dilution). Cells were acquired using a BD Biosciences LSR 4‐laser cytometer and analyzed using FlowJo. Antibody binding was quantified as the percentage of GFP‐positive cells that were also positive for IgG or IgA. Subject‐specific total IgG or IgA levels were not measured and therefore were not used to normalize the binding signals. As a result, the reported percentage binding values reflect both antigen‐specific binding and potential inherent interindividual differences in total immunoglobulin levels.

### 2.4 Detection of SARS‐CoV‐2 Anti‐N Antibody Response

Anti‐N antibody response was measured using Elecsys anti‐SARS‐CoV‐2 chemiluminescent immunoassay (Roche) following the manufacturer's instructions. The results with cut‐off index (COI) < 1.0 were defined as negative, and COI ≥ 1.0 were defined as positive.

### 2.5 Modeling Antibody Dynamics

We employed an exponential growth and decay model to quantify the antibody dynamics. The following equations describe booster‐ and infection‐elicited antibody dynamics for the breakthrough infection group (Equation 1) and uninfected group (Equation 2):

(1) $$\frac{\mathrm{dA}\left(t\right)}{\mathrm{dt}}=\left\{\begin{array}{ll} k_{1}A\left(t\right) & \left(t\leq \varphi \right)\\ -k_{2}A\left(t\right) & \left(\varphi <t\leq \tau_{i}\right)\\ k_{3}A\left(t\right) & \left(\tau_{i}<t\leq \tau_{i}+\varphi \right)\\ -k_{4}A\left(t\right) & \left(t>\tau_{i}+\varphi \right) \end{array}\right..$$

(2) $$\frac{\mathrm{dA}\left(t\right)}{\mathrm{dt}}=\left\{\begin{array}{l} \begin{array}{cc} k_{1}A\left(t\right) & \left(t\leq \varphi \right)\\ -k_{2}A\left(t\right) & \left(t>\varphi \right) \end{array} \end{array}\right..$$

Here, A(t) is the binding antibody response (%) at time t (in days), hence $A_{0}$ represents the binding antibody responses on the day of booster vaccination (i.e., $A_{0}=A(0)$). The parameters $k_{1}$ and $k_{2}$ represent the growth and decay rates of antibodies after booster vaccination, respectively. The time interval from booster vaccination or breakthrough infection to peak binding antibody response, φ, was fixed at 21 days following previous studies [15, 17, 38, 39, 40, 41, 42]. For the breakthrough infection groups, the parameters $k_{3}$, $k_{4}$, and $\tau_{i}$ are the growth and decay rates of antibodies after breakthrough infection, and the time interval between booster vaccination and breakthrough infection for individual i. A model schematic is summarized in Figure 1B.

### 2.6 Model Fitting and Characteristics of Antibody Dynamics

We fitted the model above using a nonlinear mixed‐effect approach and jointly estimated the parameters: $A_{0}$, $k_{1}$, $k_{2}$, $k_{3}$, $k_{4}$. Individual characteristics, such as age group ( < 60 years or ≥ 60 years), sex (male or female), booster vaccination type (Pfizer or Moderna), and timing of infection (early infection, late infection, or uninfected) were considered as potential covariates. Each model parameter is composed of both fixed and random effects. The fixed effect is constant across individuals, representing the average population value of the parameter, whereas the random effect reflects the interindividual variability. Specifically, the parameter set for individual i, $\theta_{i}$, is the product of the fixed effect $\theta_{\mathrm{pop}}$ and the random effect $\pi_{i}$, where the random effect is assumed to follow a normal distribution: $\pi_{i}\sim N(0,\Omega )$. We employed a nonlinear mixed‐effects modeling approach to robustly estimate parameters from limited data. In particular, estimating the parameter $k_{4}$ is especially challenging for individuals infected near D360. When using conventional maximum likelihood estimation (MLE) that relies solely on individual‐level data, the limited number of observations after infection leads to unstable or non‐identifiable estimates for $k_{4}$. The mixed‐effects model addresses this issue by assuming that individual‐level parameters are drawn from a distribution centered around a population‐level (fixed) effect. This hierarchical structure enables the model to borrow strength across participants, allowing for more stable parameter estimation even in cases with sparse data. Fixed effects and random effects were estimated via the stochastic approximation expectation‐maximization (SAEM) algorithm and empirical Bayes estimation (EBE), respectively, using Monolix 2023R1 (https://www.lixoft.com). Covariates were selected using a backward selection approach; specifically, starting from the full model where all the covariates were considered, we reduced the model covariates to minimize the Akaike information criterion (AIC).

To intuitively understand the impact of individual characteristics on antibody dynamics, we performed a series of univariable and multivariable linear regressions using the antibody levels at three different time points as outcomes: (i) the measured D28 binding antibody levels, (ii) peak binding antibody response after booster vaccination, and (iii) peak binding antibody response after breakthrough infection (only for those infected during the follow‐up period). Note that outcomes (ii) and (iii) were computed for each individual using the best‐fit individual parameters. Similarly, individual characteristics, such as age group, sex, booster type, and timing of infection were considered as predictors. Figure 1B shows a visual representation of modeled antibody dynamics and the outcomes.

### 2.7 Association Between Antibody Levels and SARS‐CoV‐2 Infection Risk and Predictions of Protection

To assess the association between binding antibody levels and risk of SARS‐CoV‐2 infection, we performed survival analyzes with Cox proportional hazards (PH) regression models using antibody levels as the main predictors and the infection event as the outcome. Individuals who remained uninfected were treated as censored. Censoring occurs when information about the outcome for some individuals is unknown. In this case, individuals who did not get infected during the follow‐up period are considered censored because whether they later became infected or not is unknown. We accounted for this by marking their data as incomplete rather than excluding it. We performed survival analyzes separately for each antibody isotype (i.e., WT IgG, WT IgA, BA.1 IgG, and BA.1 IgA), and considered the following as covariates: age group, sex, booster type, and mean daily COVID‐19 incidence for each week in Singapore (obtained from a COVID‐19 data repository [43]).

Continuous antibody levels were used as main predictors in two ways. First, we used the D28 antibody levels, which was considered as the booster response measurement after antibody levels had reached their postvaccination peak. Note that none of the participants were infected before D28. Second, we used the daily antibody levels estimated from our fitted models (with the best‐fit parameters) as time‐varying covariates to represent the dynamic nature of binding antibody responses across time [44].

Additionally, we performed the same survival analyzes using categorical antibody levels. We divided the antibody levels into three groups using all the estimated antibody values after the booster peak till the end of the follow‐up period for each antibody isotype: (1) at or below the 25th percentile (“low”), (2) between the 25th‐75th percentiles (“medium”), and (3) at or above the 75th percentile (“high”). We used the AIC and Bayesian information criterion (BIC) to compare the prediction ability of the survival models with booster response and time‐varying antibody levels.

Using the results from the Cox PH models, we estimated the chance of protection, defined as the percent chance of not being infected by SARS‐CoV‐2. The analysis focused on evaluating the levels of protection in relation to BA.1 IgA binding antibody levels and the time since receiving the booster vaccination. Three incidence scenarios—“low,” “medium,” and “high”—were defined based on Singapore's COVID‐19 case counts during the follow‐up period [43]. These correspond to the first quartile (379 cases per million per day), median (621 cases per million per day), and third quartile (1157 cases per million per day) of average daily reported cases, respectively.

## 3 Results

### 3.1 Participant Characteristics and Antibody Levels

In this study, we studied a cohort of adult individuals in Singapore which was recruited as part of the PRIBIVAC study, a randomized controlled subject‐blinded trial to evaluate the immunogenicity and safety of heterologous booster vaccinations compared to homologous booster vaccinations. We analyzed the antibody dynamics of 86 individuals, aged 21 to 84 years (median of 54.5), who were closely monitored for up to 360 days following booster vaccination. A total of 38 (44.2%) participants were male, 43 (50.0%) received a Pfizer booster vaccine (the rest received Moderna), and 77 (89.5%) were Chinese. During the follow‐up period, 59 (68.6%) participants experienced breakthrough infection, of which 29 (49.2%) participants became infected within the first 180 days. However, there were no statistically significant differences between the proportion of uninfected and infected participants by age, sex, booster type, or ethnicity (Table 1).

**Table 1:** Summary of participant demographics.

Variables	Total (*N* = 86)	Breakthrough infection during follow‐up		
		No (*N* = 27, 31.4%)	Yes (*N* = 59, 68.6%)	*p* value^a^
Age (median, IQR)	54.5 (31.5, 65.0)	45.0 (31.0, 65.5)	58.0 (34.5, 64.0)	0.830
Age group (*N*, %)				
< 60 years old	47 (54.7)	16 (59.3)	31 (52.5)	0.561
≥ 60 years old	39 (45.3)	11 (40.7)	28 (47.5)	
Sex (*N*, %)				
Female	48 (55.8)	16 (59.3)	32 (54.2)	0.663
Male	38 (44.2)	11 (40.7)	27 (45.8)	
Booster type (*N*, %)				
Moderna	43 (50.0)	14 (51.9)	29 (49.2)	0.816
Pfizer	43 (50.0)	13 (48.1)	30 (50.8)	
Timing of infection^b^ (*N*, %)				
Early infection	29 (33.7)	NA	29 (49.2)	NA
Late infection	30 (34.9)	NA	30 (50.8)	
Ethnicity (*N*, %)				
Chinese	77 (89.5)	24 (88.9)	53 (89.8)	0.469
Malay	2 (2.33)	0 (0)	2 (3.39)	
Indian	4 (4.65)	1 (3.70)	3 (5.08)	
Others	3 (3.49)	2 (7.41)	1 (1.69)	
Days from first dose of primary vaccination to booster dose (median, IQR)	266 (229, 282)	271 (253, 289)	261 (228, 280)	0.133
Days from booster dose to infection (median, IQR)	181 (120, 251)	NA	181 (120, 251)	NA

Abbreviation: IQR, interquartile range.

^a^ Comparison between uninfected and infected participants; by Mann–Whitney *U* test for continuous variables and chi‐square test or Fisher's exact test (for any expected counts < 5) for categorical variables.

^b^ Early infection: infected between D28 and D180; late infection: infected between D180 and D360.

* *p* < 0.05

** *p* < 0.01

*** *p* < 0.001.

We then stratified the raw antibody trends over the follow‐up period for each antibody isotype by the timing of infection (Figure 1C). Overall, early infections boosted the antibody levels at D180, while antibody levels continued to decline in late infection group until the occurrence of infections after D180. These antibody levels are also summarized in Table 2. Based on the Kruskal‐Wallis test, WT IgG and BA.1 IgA antibody levels were significantly different among participants who were infected early, infected late, or remained uninfected (Table 2). A post‐hoc Dunn's test (Bonferroni‐adjusted) revealed that the late infection group had significantly lower WT IgG (*p* = 0.02) and BA.1 IgA (*p* = 0.005) binding antibodies on D28 compared with uninfected participants. However, the early infection group only had significantly lower WT IgG (*p* = 0.03) binding antibodies on D28 compared with the uninfected group (Table 2).

**Table 2:** Summary of binding antibody levels (%) at D0, D28 (booster response), D180, and D360^a^.

Antibody isotype	All participants	Categorical antibody levels^b^			Timing of infection^c^			
		Low	Medium	High	Uninfected	Early infection	Late infection	*p* value^d^
**WT IgG**								
D0	19.8 (12.6, 25.9)	19.8 (12.6, 25.9)	NA	NA	20.0 (12.8, 25.6)	22.5 (10.8, 26.9)	19.5 (13.3, 24.0)	0.741
D28	50.2 (47.9, 53.1)	41.5 (39.7, 41.7)	48.5 (47.4, 49.6)	53.8 (52.2, 55.4)	52.1 (50.0, 55.1)^a^	49.1 (47.6, 52.5)^b^	49.5 (47.5, 52.1)^b^	0.010**
D180	49.2 (40.8, 55.2)	37.5 (29.4, 41.5)	45.9 (44.6, 49.2)	55.8 (53.9, 59.3)	43.9 (37.4, 51.3)	57.7 (55.2, 59.5)	42.0 (37.0, 44.9)	NA
D360	53.8 (47.8, 57.9)	29.3 (22.0, 37.1)	47.8 (45.7, 49.0)	57.0 (53.9, 60.0)	44.7 (29.3, 49.4)	53.9 (49.7, 56.2)	57.8 (55.4, 60.8)	NA
**WT IgA**								
D0	0.8 (0.5, 2.6)	0.8 (0.4, 2.5)	23.5 (23.5, 23.5)	NA	0.8 (0.5, 3.8)	1.0 (0.6, 2.6)	0.8 (0.4, 1.2)	0.230
D28	35.5 (27.2, 41.4)	19.7 (19.0, 20.7)	31.7 (26.6, 34.1)	42.7 (39.5, 45.5)	34.1 (29.5, 39.7)	36.3 (29.7, 42.7)	35.1 (25.2, 41.4)	0.938
D180	30.3 (16.9, 43.6)	12.8 (8.7, 17.5)	30.1 (26.8, 32.9)	45.1 (43.0, 51.3)	21.5 (14.6, 31.0)	45.3 (43.1, 51.8)	22.3 (11.0, 29.8)	NA
D360	37.2 (28.6, 46.0)	11.6 (4.6, 14.1)	33.0 (30.3, 34.3)	46.1 (40.9, 49.6)	12.8 (6.3, 32.5)	37.9 (33.0, 45.6)	45.4 (39.2, 51.5)	NA
**BA.1 IgG**								
D0	3.1 (1.7, 4.9)	3.1 (1.7, 4.9)	32.2 (32.2, 32.2)	NA	3.6 (1.9, 5.3)	2.7 (1.6, 5.4)	3.0 (1.8, 3.9)	0.705
D28	41.9 (34.3, 46.7)	19.0 (19.0, 19.0)	33.5 (29.0, 37.5)	46.0 (42.9, 48.5)	44.0 (37.7, 47.2)	42.9 (33.3, 46.7)	39.1 (33.4, 44.1)	0.168
D180	30.6 (12.1, 48.8)	8.2 (5.7, 18.3)	31.2 (27.2, 34.6)	54.6 (47.8, 58.5)	25.7 (10.4, 31.6)	54.8 (49.1, 58.7)	16.6 (7.3, 27.4)	NA
D360	43.2 (28.3, 50.8)	7.3 (4.5, 12.6)	37.2 (29.6, 38.6)	49.9 (450, 54.1)	16.8 (6.2, 33.7)	45.7 (39.7, 50.8)	50.0 (43.5, 54.5)	NA
**BA.1 IgA**								
D0	1.0 (0.7, 1.6)	1.0 (0.6, 1.5)	9.4 (7.7, 11.1)	NA	0.9 (0.6, 1.5)	1.2 (0.8, 1.7)	0.9 (0.6, 1.3)	0.241
D28	19.8 (15.5, 25.8)	3.7 (3.7, 3.7)	12.8 (9.2, 15.7)	24.2 (20.5, 28.6)	24.7 (18.3, 29.1)^a^	19.6 (15.5, 21.9)^a^, ^b^	17.7 (10.5, 21.8)^b^	0.006**
D180	7.0 (1.8, 34.5)	1.6 (1.2, 2.3)	7.2 (6.6, 9.9)	40.6 (31.2, 47.0)	3.4 (1.9, 7.0)	44.4 (34.8, 47.1)	1.8 (1.2, 4.4)	NA
D360	20.8 (5.8, 31.7)	0.8 (0.6, 2.0)	9.0 (5.8, 15.1)	30.0 (25.2, 35.3)	2.8 (0.7, 7.1)	24.8 (17.6, 29.9)	32.4 (23.4, 38.3)	NA

^a^ Numbers are median and interquartile range (in parentheses).

^b^ Using all estimated antibody levels after booster peak till the end of the follow‐up period for each antibody isotype: the 25th percentile (“low”), in the 25th–75th percentiles (“medium”), and at or above the 75th percentile (“high”).

^c^ Early infection: infected between D28 and D180; late infection: infected between D180 and D360; uninfected: no infection during the period of follow‐up.

^d^ Comparison among different timings of infection by the Kruskal‐Wallis test and Dunn's post hoc test on D0 and D28 (not performed on D180 and D360 as antibody level is influenced by infection); groups sharing a common letter were not significantly different (by Dunn's test).

* *p* < 0.05

** *p* < 0.01

*** *p* < 0.001.

### 3.2 Estimated Antibody Dynamics Model

To understand the dynamics of SARS‐CoV‐2 antibody levels post‐booster vaccination to inform durations of protection, we mapped the antibody kinetics for IgA and IgG binding against WT and BA.1 S proteins. The observed antibody trends—namely, boosting after vaccination or infection followed by gradual waning—motivated the structure of our model. We incorporated parameters to capture both the rapid increase in antibody levels after immune stimulation (vaccination or infection) and the slower decline over time. This model structure enables the reconstruction of individual‐level antibody trajectories, even in participants with sparse sampling, by leveraging on population‐level dynamics. These reconstructed trajectories form the basis for our predictive framework, as they allow us to treat antibody levels as time‐varying covariates in survival analyzes to quantify their association with infection risk.

**Figure 3:**
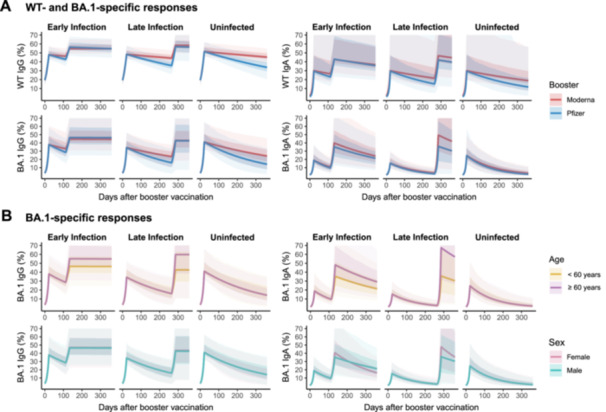
Predicted longitudinal antibody trajectories. The antibody trajectories were drawn by running the mathematical model using the estimated population parameters, stratified by the infection status (early infection, late infection, and uninfected). The timing of infection was fixed at the mean (Day 112 for early infection and Day 264 for late infection). All other parameters were fixed at the reference category: < 60 years, male, Pfizer. (A) The effect of booster type (Pfizer or Moderna) on WT‐ and BA.1‐specific responses. (B) The effect of age group ( < 60 years or ≥ 60 years) and sex (male or female) on BA.1‐specific responses. For WT‐specific responses, age group and sex were excluded during covariate selection and thus not incorporated into the simulations. Solid lines represent predicted antibody levels based on the best‐fit population parameters. Darker shaded areas indicate the 50% prediction interval, while lighter shaded areas denote the 90% prediction interval, both derived from 1000 randomly sampled sets of model parameters.

Using a nonlinear mixed‐effects model, we estimated antibody dynamics and identified key predictors of post‐booster and postinfection immune responses. Estimated antibody trajectories for each individual are shown in Supporting information Figures S1–S4, while Figure 3 illustrates the model‐based trajectories for populations with specific characteristics, generated using the estimated population parameters (Supporting information Tables S1–S4). It is important to note that Figure 3 represents the predicted “typical” antibody dynamics rather than observed individual data. For visualization purposes, the timing of infection in Figure 3 was set hypothetically, using the mean infection timing for the early and late infection groups. For WT IgG (Supporting information Table S1), WT IgA (Supporting information Table S2), and BA.1 IgG (Supporting information Table S3), we found that the decay rates of antibodies after booster vaccination ($k_{2}$) and the growth rates of antibodies after infection ($k_{3}$) were significantly higher in those who received a Pfizer booster compared to Moderna. For BA.1 IgG and BA.1 IgA (Supporting information Table S4), the growth rates of antibodies after infection ($k_{3}$) were higher in the elderly group (≥ 60 years) and slightly higher in females, compared to the younger group (< 60 years) and males. Furthermore, the growth rates of antibodies after infection ($k_{3})$ were higher in the late infection group than the early infection group for all antibody isotypes, which might be explained by lower antibody levels at the time of infection for the late infection group.

**Figure 4:**
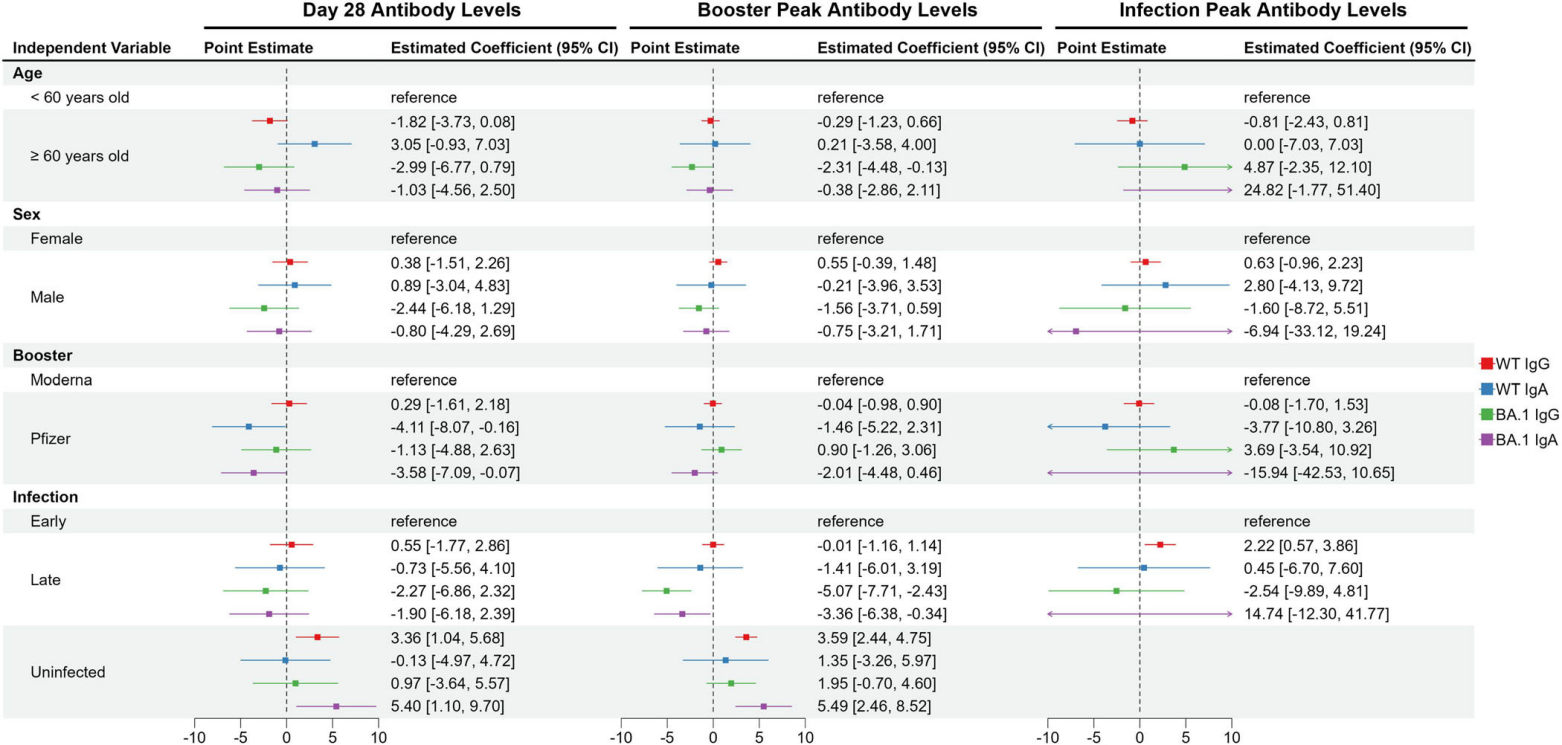
Association between participant characteristics and antibody levels. Results of the multivariable linear regression analyzes using age, sex, booster type, and timing of infection for each participant as predictors, with the following outcomes: (i) measured D28 binding antibody levels, (ii) peak binding antibody response after booster vaccination, and (iii) peak binding antibody response after breakthrough infection (for those infected during the follow‐up period). Point estimates of the regression coefficients are listed along with 95% confidence intervals (CIs) in parentheses, with each antibody isotype represented by different colors.

The multivariable linear regression analyzes reveal that, for WT IgG and BA.1 IgA, both the peak binding antibody response following booster vaccination and the D28 antibody responses were higher in the uninfected group compared to the early infection group (Figure 4). Interestingly, for BA.1 IgG and IgA, the antibody peaks after booster vaccination were lower in the late infection group compared to the early infection group. Moreover, those who received a Pfizer vaccine as a booster showed a lower D28 antibody response for WT IgA and BA.1 IgA. Univariable analyzes largely supported the results of multivariable analysis, with the exception that the peak binding antibody response of BA.1 IgA following breakthrough infection was significantly higher in the elderly group ( ≥ 60 years) (Supporting information Figure S5).

### 3.3 Antibodies as Correlates of Protection and Their Duration of Protection

**Figure 5:**
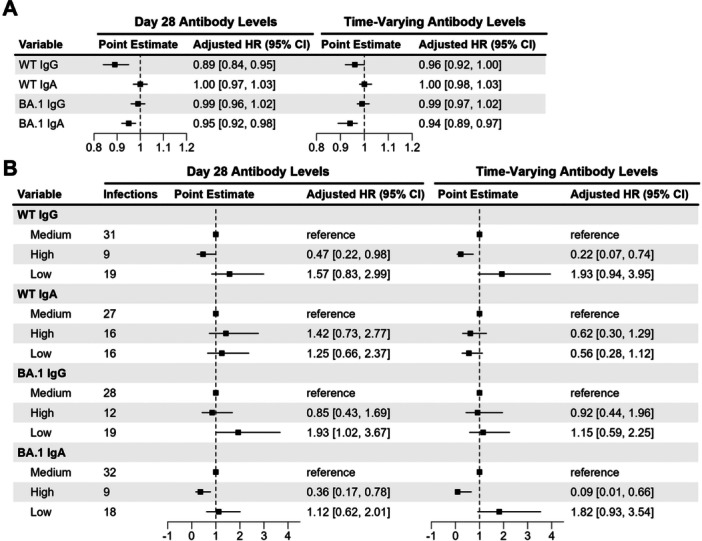
Risks of SARS‐CoV‐2 infection by antibody isotype. Adjusted hazard ratios (HRs) and their 95% confidence intervals (CIs) are depicted for antibody levels treated as (A) continuous variables and (B) categorical variables within the Cox proportional‐hazards models. Both antibody levels measured on Day 28 (booster response) and time‐varying antibody levels were considered. Categorical antibody levels were divided into three groups using all the estimated antibody values after the booster peak till the end of the follow‐up period for each antibody isotype: (1) at or below the 25th percentile (“low”), (2) between the 25th–75th percentiles (“medium”), and (3) at or above the 75th percentile (“high”).

We evaluated antibody levels as correlates of protection against SARS‐CoV‐2 infection and estimated the duration of protection using survival models. We first performed survival analyzes using continuous antibody levels (%). After adjusting for age, sex, booster type, and COVID‐19 incidence, HRs for booster response binding antibody levels were 0.89 (95% CI [0.84, 0.95]) for WT IgG and 0.95 (95% CI [0.92, 0.98]) for BA.1 IgA for each percent increase in binding antibodies, respectively (Figure 5A). In other words, every 1% reduction in antibody levels for WT IgG and BA.1 IgA corresponds to 11%‐ and 5%‐fold increase in the risk of infection, respectively. Similarly, using time‐varying antibody levels as the predictors, where booster response antibody levels are updated daily based on our fitted models, the HR was 0.94 (95% CI [0.89, 0.97]) for BA.1 IgA (Figure 5A). Notably, the WT IgG was no longer significant when treated as time‐varying covariates, with a HR of 0.96 (95% CI [0.92, 1.00]). All other HRs from the survival models using continuous antibody levels (booster response and time‐varying) as predictors are depicted in Figure 5A.

We used the AIC and BIC to compare the prediction ability of the survival models with booster response or time‐varying antibody levels, respectively. Note that these metrics were only computed for WT IgG and BA.1 IgA as they are significant infection risk factors. For both WT IgG and BA.1 IgA, AIC and BIC were consistently lower for the Cox models treating binding antibody levels as time‐varying variables compared to the models using solely booster response binding antibody levels, suggesting that time‐varying antibody levels have higher performance in predicting infection risk (Table 3).

**Table 3:** Model comparison metrics calculated for survival models.

Antibody isotype	Predictor			
	D28 antibody levels (booster response)		Time‐varying antibody levels	
	AIC	BIC	AIC	BIC
WT IgG	469.94	480.33	457.05	467.44
BA.1 IgA	471.95	482.34	452.76	463.15

Next, we performed the same analyzes using categorical antibody levels (i.e., low, medium, and high). Using categorical D28 binding antibody responses (booster response) as predictors (see Table 2 for antibody levels for each category), we found that high WT IgG and BA.1 IgA levels were associated with lower risk of infection (HRs: 0.47 (95% CI [0.22, 0.98]) and 0.36 (95% CI [0.17, 0.78])) compared to the medium antibody levels (Figure 5B). In addition, low D28 BA.1 IgG binding responses significantly increased the risk of infection with a HR of 1.93 (95% CI [1.02, 3.67]) compared to the medium antibody level. We observed similar trends when using categorical time‐varying antibody responses as predictors. High WT IgG levels and high BA.1 IgA levels were associated with lower risk of infection compared with the medium level, which corresponding HRs of 0.22 (95% CI [0.07, 0.74]) and 0.09 (95% CI [0.01, 0.66]), respectively (Figure 5B).

**Figure 6:**
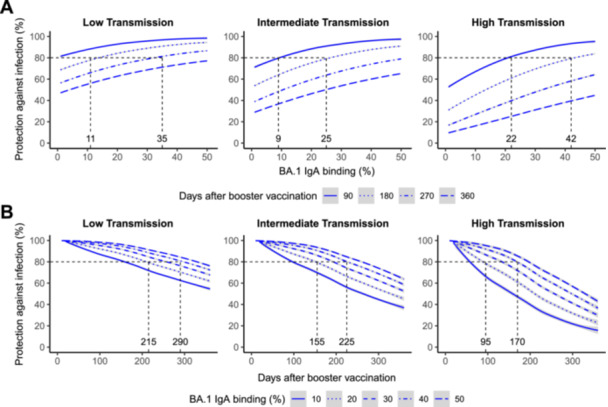
Predicted protection durations against SARS‐CoV‐2 infection. Predicted durations of protection against infection (%) are plotted as functions of (A) binding antibodies against the BA.1 IgA spike (%), and (B) days since booster vaccination. The darker shaded areas represent the 95% confidence bands of the smoothed function. Three different incidence scenarios are plotted based on Singapore's reported COVID‐19 case counts during the follow‐up period: (1) “low transmission” was set as having a mean of 379 cases per million per day, (2) “medium transmission” as 621 cases per million per day, and (3) “high transmission” as 1157 cases per million per day. The predicted BA.1 IgA spike binding (%) required to achieve ≥ 80% protection against infection at 3 and 6 months (for low transmission) as well as 6 and 9 months (for medium and high transmission) are outlined with the black dotted lines in (A). The predicted number of days after booster vaccination that confers ≥ 80% protection assuming that BA.1 IgA binding wanes to 20% and 40% are outlined with black dotted lines in (B).

Utilizing our estimated survival probabilities derived from the Cox PH models, we predicted the protection brackets, focusing on having at least 80% protection against infection. In other words, we predicted the level of binding antibodies such that 8 out of 10 individuals would remain uninfected, and we predicted how long such protection would last. Under low COVID‐19 incidence, achieving at least 80% protection for 6 to 9 months against SARS‐CoV‐2 infection requires 11%–35% BA.1 IgA binding antibody levels (Figure 6A). If the booster vaccination antibody response reaches 20%–40% BA.1 IgA binding, this level of protection can be maintained for 215 to 290 days (Figure 6B). Under medium incidence, 9%–25% binding levels are needed for the same level of protection for 3 to 6 months, with 20%–40% binding providing protection for 155 to 225 days (Figure 6). In high incidence scenarios, 22%–42% binding is required for 3 to 6 months of protection, while 20%–40% binding results in at least 80% protection for 95 to 170 days (Figure 6).

## 4 Discussion

Using longitudinal antibody data collected from individuals in Singapore, we developed a mathematical model that describes the dynamic change in antibody levels considering the effects of waning, type of booster vaccination, and SARS‐CoV‐2 infection. We found that Pfizer vaccines (compared with Moderna vaccines), female individuals, older age, and late infection (compared with early infection) were associated with a stronger immune response after breakthrough infection for at least some antibody isotypes. The Pfizer vaccine also shows an accelerated decay after booster vaccination for WT IgG/IgA and BA.1 IgG. Previous studies have also found that SARS‐CoV‐2 anti‐S antibody titers are more elevated with heterologous vaccination (2 Pfizer and 1 Moderna dose) compared to homologous vaccination (3 Pfizer doses), and IgG antibody responses were specifically shown to be significantly better [45, 46]. Additionally, the peak binding antibody levels following booster vaccination were higher in the uninfected group compared to the early infection group for WT IgG and BA.1 IgA. On D28, antibody levels were higher in the uninfected group (compared to the infected group) and in those with Moderna (compared to Pfizer) vaccinations. We further examined the association between antibody levels and infection risk through survival analyzes using either booster response or time‐varying antibody levels as predictors. Overall, higher IgA binding against the BA.1 spike was associated with higher protection against infection, and the time‐varying antibody levels as predictors resulted in better‐fit models, suggesting the importance of monitoring antibody responses over time. In a setting with a medium level of COVID‐19 incidence (621 cases per million per day), an antibody response of 20%–40% BA.1 IgA binding antibodies is needed to achieve at least 80% protection against infection for an average of 155–225 days within our study cohort.

Our multivariable regression analysis revealed that peak and D28 titers of WT IgG and BA.1 IgA were higher in the uninfected group compared to the early infection group, with the baseline levels being similar between the different groups. We also observed that the booster vaccination containing the WT S protein elicited a robust homologous response, particularly in WT IgG and IgA levels. In fact, WT IgA responses are similar between the different groups. This suggests that the booster vaccination is effective in enhancing immune responses against homologous antigens. Our multivariable regression analysis also revealed that only BA.1 IgA, but not WT IgA, responses after the booster vaccination, were lower in the late infection group, suggesting that this is a variant‐specific effect, in this case, BA.1. Taken together, these findings suggest that individuals with higher post‐booster antibody levels, particularly variant‐specific antibodies, may have been less likely to become infected, suggesting a potential protective role of humoral responses. While our study was not designed to establish causality, these interpretations underscore the complexity of the relationship between antibody kinetics and infection risk, and highlight the need for further investigation, including analyzes incorporating other immunological biomarkers such as T cell and B cell responses.

Our findings that IgG antibodies against WT and IgA antibodies against the BA.1 Omicron variant are correlated with protection have significant implications for future vaccine design and immunization strategies. Specifically, these results suggest that developing vaccines that induce robust IgA responses may enhance protection against emerging Omicron subvariants. Furthermore, the correlation between antibody levels and protection can inform the optimal frequency of booster vaccinations. Optimizing the timing of booster vaccines could help achieve a desired level of protection while minimizing the economic and logistical burdens of repeated vaccination. Our study underscores the importance of targeted immunological responses in vaccine development and the need for adaptive vaccination strategies to combat evolving viral threats.

Individual characteristics associated with antibody trajectories, such as age and sex, were also identified in the present study. The antibody responses of elderly and female individuals increased more quickly after infection compared to male and younger individuals, which might be explained by the other biological differences beyond just antibodies. For example, the higher antibody response in females can be attributed to the interaction between sex hormones and immune cells [47, 48, 49]. This highlights the complex interplay of biological age‐ and sex‐specific factors in shaping immune responses and underscores the need for a comprehensive approach to vaccination strategies. Further studies are needed to understand the biological mechanisms creating such differences in individual antibody trajectories.

While we focused on serum antibody responses, it is important to recognize that other arms of the immune system—particularly mucosal IgA and T‐cell immunity—also play key roles in protection against SARS‐CoV‐2. Mucosal IgA serves as a frontline defense at the site of viral entry, potentially preventing initial infection or reducing viral replication in the upper respiratory tract [50, 51]. Likewise, T cell responses contribute to viral clearance and have been shown to provide protection even when circulating antibody levels wane or are low [52, 53]. Future work integrating mucosal and cellular immunity into the data collection and modeling frameworks will be essential to more comprehensively characterize immune protection and inform vaccine design and evaluation.

This study possesses several notable strengths. One of the primary strengths is the longitudinal measurement of binding antibody responses at multiple critical timepoints. Blood samples were collected on the day of the booster vaccination and at 28, 180, and 360 days afterward. This design allows for a comprehensive analysis of long‐term immune responses, providing valuable insights into antibody dynamics over time. Second, the date of infection is known, which enabled us to model the antibody dynamics before and after breakthrough infections. These temporal data allowed us to discern significant differences in antibody growth rates after infection, particularly highlighting variations based on age, sex and booster type. Third, our model is sufficiently robust to fully reconstruct the antibody levels post‐booster vaccination and infection from limited datapoints. This capability enhances the study's ability to predict long‐term immune responses. Finally, our study measured four antibody isotypes simultaneously: binding IgG and IgA against both the WT and Omicron variant BA.1 S proteins.

However, there are a few limitations that need to be noted. First, the exposure of each individual to the virus is unknown, which may influence the risk of infection. Different individuals may have closer and/or more frequent contact with COVID‐19 patients. Nonetheless, we accounted for the possible differences in exposure throughout the study period in our survival models by adjusting for the weekly incidence of COVID‐19 cases in Singapore as a time‐varying covariate. Second, we performed separate survival models for each antibody isotype, but we did not employ combined models where different antibody isotypes, such as WT IgA and IgG, were considered together as predictors within the same model. Our focus was to evaluate the unique contribution of each antibody isotype against the risk of infection by analyzing each type separately. In addition, separate analyzes can minimize the risk of multicollinearity or other statistical issues that may arise when combining potentially highly correlated variables within a model; several studies lack this separation of specific antibody isotypes and can be referenced to find the effects of combined antibody isotypes [8, 11, 54]. Third, majority of the cohort is Chinese, thus the analysis could have benefitted from having more samples collected from individuals with diverse backgrounds to improve the predictability of the model. While the sample size was relatively small, the nonlinear mixed‐effects models effectively leveraged repeated measures to improve statistical power and estimation accuracy by including interindividual variability as random effects. Fourth, only serum antibodies were analyzed in this study, and antibodies found in the nasal cavity were not measured, which may be more relevant in preventing infection. Fifth, we used the SFB assay for anti‐S antibody response measurement to capture the IgA response and did not measure neutralizing antibody (nAb) levels. Although our model is based on binding IgA measurements, several studies have reported moderate to strong correlations between IgA—particularly mucosal or spike‐specific IgA—and neutralizing activity against SARS‐CoV‐2, including Omicron variants [51, 55]. Future studies incorporating parallel measurements of nAbs would help to validate and refine the modeled protection estimates derived from binding antibody levels.

In conclusion, our study identifies BA.1 IgA and WT IgG binding antibodies as correlates of protection against SARS‐CoV‐2 infection. Additionally, we modeled the trajectory of the antibody response following mRNA vaccination and SARS‐CoV‐2 infection, which provided more detailed and numerous data points for survival analysis, thus enhancing the depth of the analysis. We emphasize the importance of measuring antibody levels over time to be able to model their trajectories more accurately. However, in addition to antibody levels, future research should consider additional factors, such as T cell responses and the cell‐mediated functions of antibodies, for a comprehensive understanding of immune protection against SARS‐CoV‐2 infection and optimization of mRNA vaccination strategies.

## Author Contributions

Y.S.G. and K.E. conceived and designed the study. Data were obtained and analyzed by L.P., Y.W., A.S., H.K.C., M.C., P.X.H., C.Y.L., Y.S.G., and K.E. The manuscript was written by L.P., Y.W., A.S., H.K.C., M.C., L.R., Y.S.G., and K.E. Cohort recruitment and sample collection were supervised and coordinated by X.Y.P., S.R., P.Y.C., S.W.X.O., T.H.L., R.J.H.L., C.L., J.T., the NCID Study Group, D.C.L., and B.E.Y. Sample processing was carried out by the COVID‐19 Cohort Study Group. All authors reviewed and approved the final article.

## NCID Study Group

Jocelyn Jin Yu, Zheng Kuang Soh, Yi Qing Chin, Jonathan Jordon Lim, Juwinda Ongko, Eshele Anak Libau, Celine Theo, Mohammed Ridzwan Bin Abdullah, Shiau Hui Diong, He Ping Yeo.

## COVID‐19 Cohort Study Group

Angeline Rouers, Chang Zi Wei, Matthew Zirui Tay, Anthony Torres‐Ruesta, Nathan Wong, Yuling Huang, Alice Soh Meoy Ong, Adeline Chiew Yen Chua, Samantha Nguee, Yong Jie Tan, Vanessa Neo, Isaac Kai Jie Kam, Ajayanandan Yadunandan, Sooriya Kannan Selvam, Jarvis Goh, Ng Kah Ying, Sim Xin Yi, Wong Wei Lun, Anna Xinyi Loo, Liang Hui Loo.

## Conflicts of Interest

The authors declare no conflicts of interest.

## Supporting information


**Figure S1:** Estimated WT IgG antibody dynamics.

**Figure S2:** Estimated BA.1 IgG antibody dynamics.

**Figure S3:** Estimated WT IgA antibody dynamics.

**Figure S4:** Estimated BA.1 IgA antibody dynamics.

**Figure S5:** Association between antibody levels and participant characteristics.

**Table S1:** Estimated model parameters for the WT IgG antibody model.
**Table S2:** Estimated model parameters for the WT IgA antibody model.
**Table S3:** Estimated model parameters for the BA.1 IgG antibody model.
**Table S4:** Estimated model parameters for the BA.1 IgA antibody model.

## Data Availability

The data that support the findings of this study are available on request from the corresponding author. The data are not publicly available due to privacy or ethical restrictions. Data that support the findings of this study are available from the corresponding authors upon reasonable request. All analyzes were performed with the statistical computing software R (version 4.3.3) (https://www.r-project.org/). The analysis using nonlinear mixed‐effects modeling was performed on Monolix 2023R1 (https://www.lixoft.com/). Code files are available at the following repository: https://github.com/SherryYuqianWang/Antibody.
