# Supplementary figures and images for: Modeling Antibody Kinetics Post‐mRNA Booster Vaccination and Protection Durations Against SARS‐CoV‐2 Infection
Source: J Med Virol. 2025 Aug 6;97(8):e70521. doi: 10.1002/jmv.70521 (PMC12327169; doi:10.1002/jmv.70521)

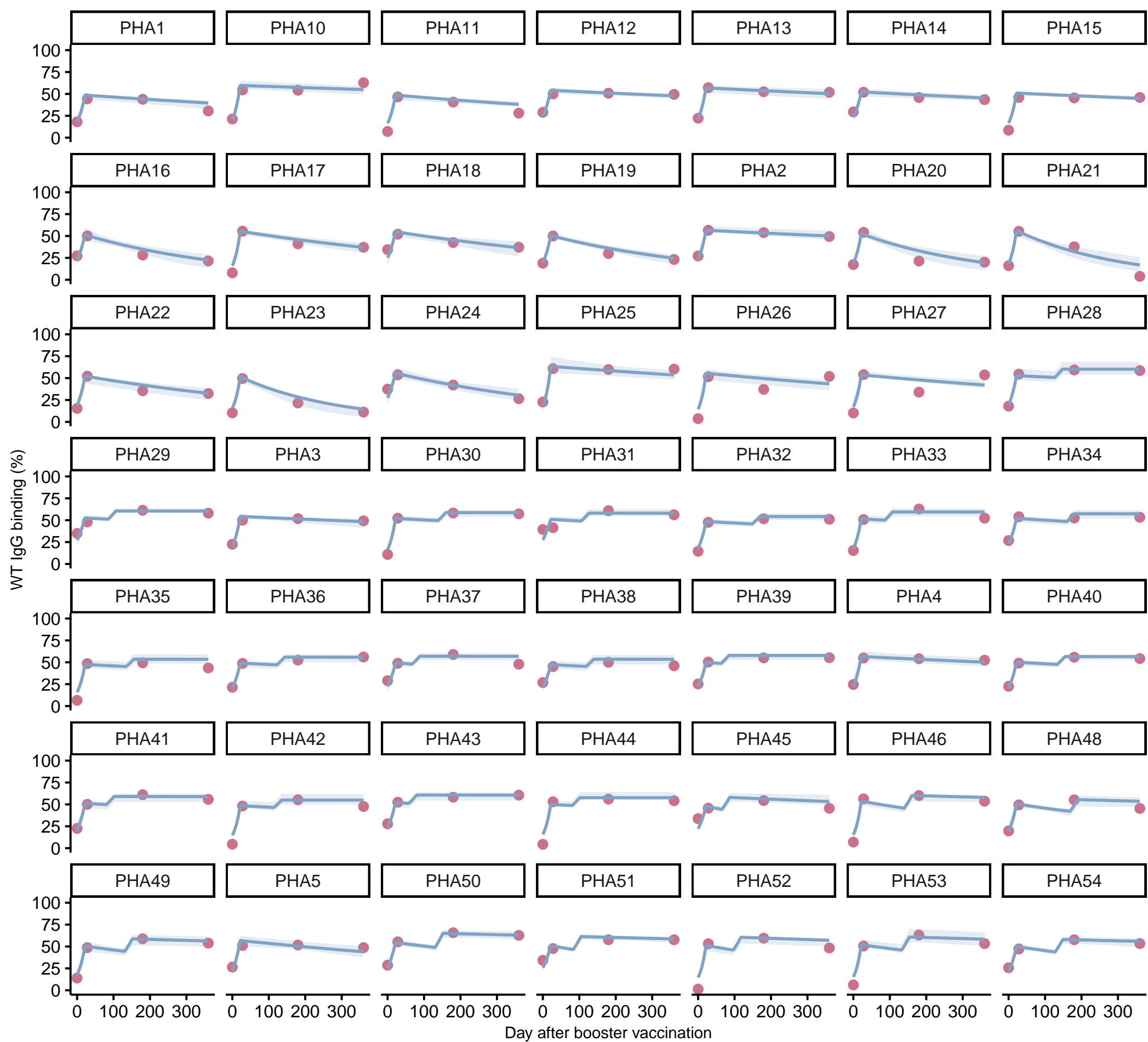

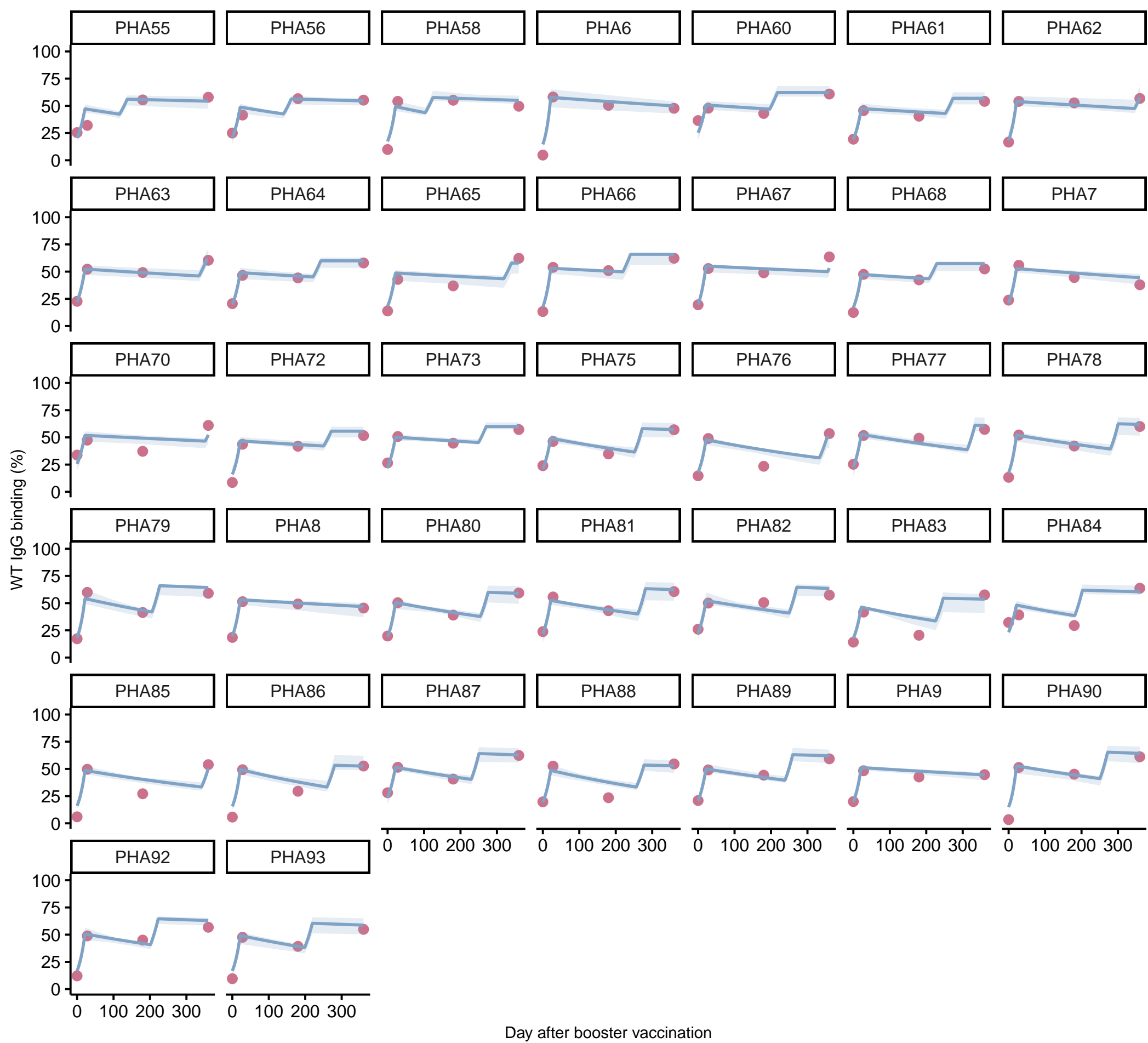

Supplement: Supplementary file 1 — Figure S1: Estimated WT IgG antibody dynamics. [file JMV-97-e70521-s003.pdf]

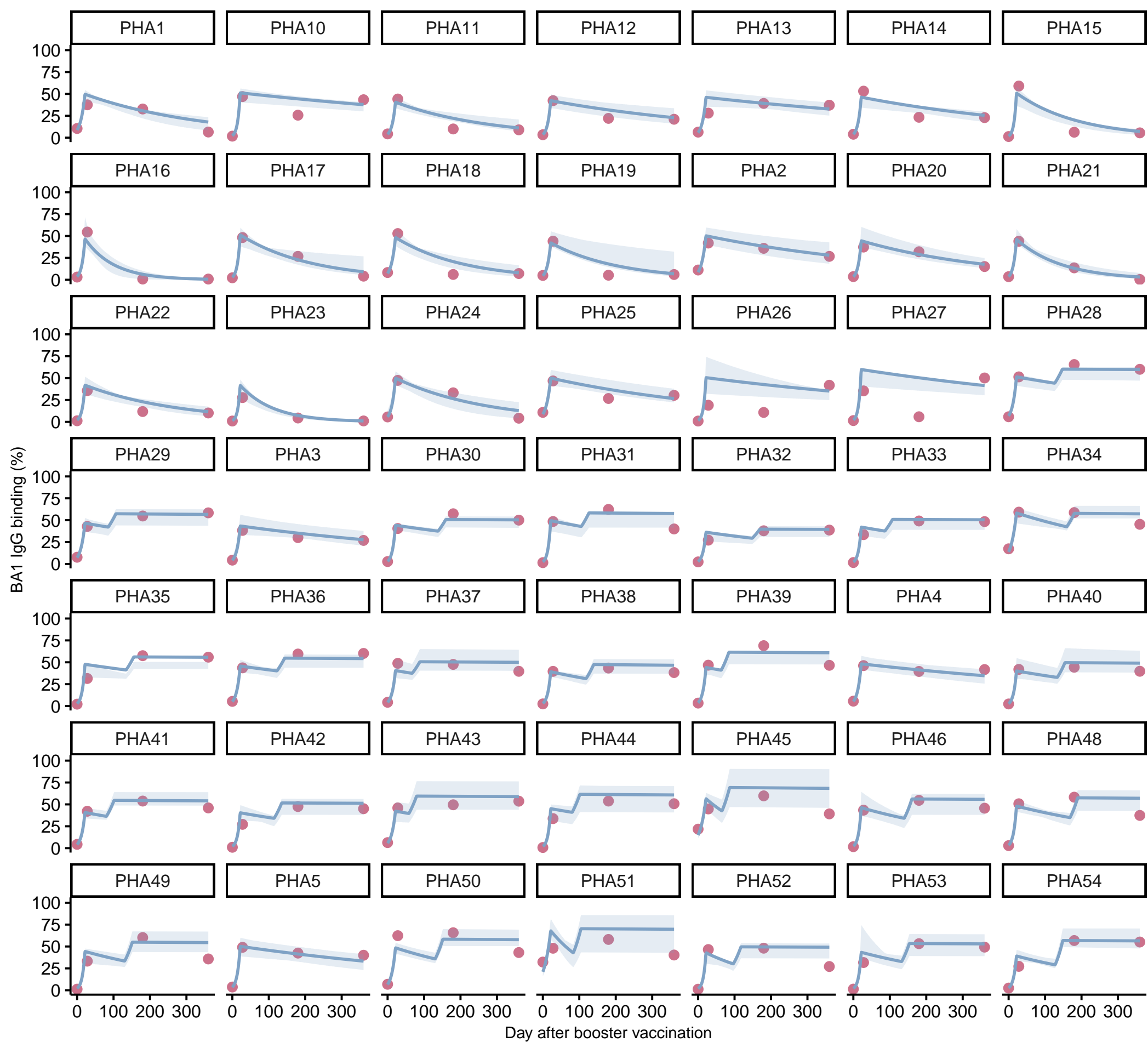

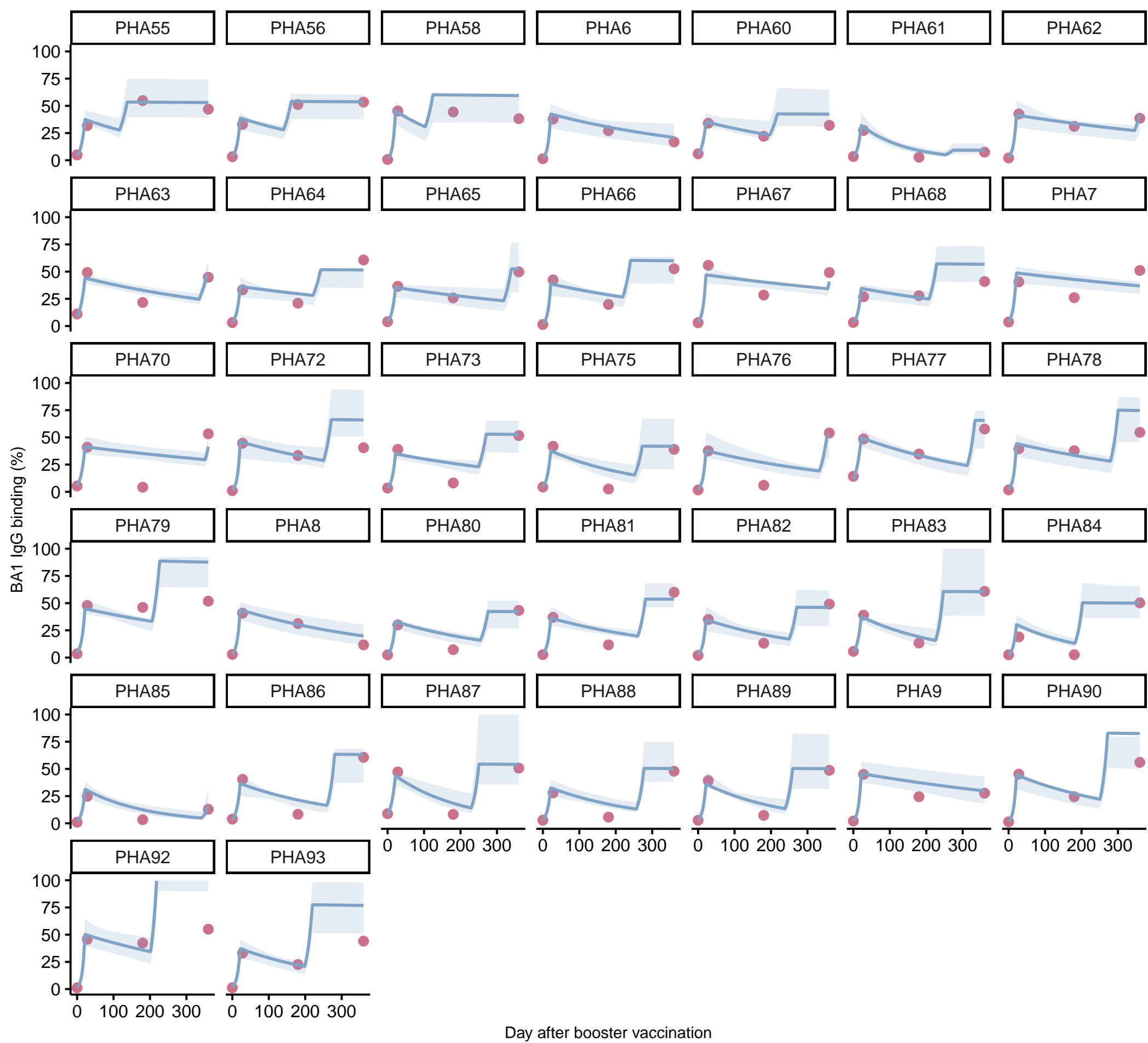

Supplement: Supplementary file 2 — Figure S2: Estimated BA.1 IgG antibody dynamics. [file JMV-97-e70521-s004.pdf]

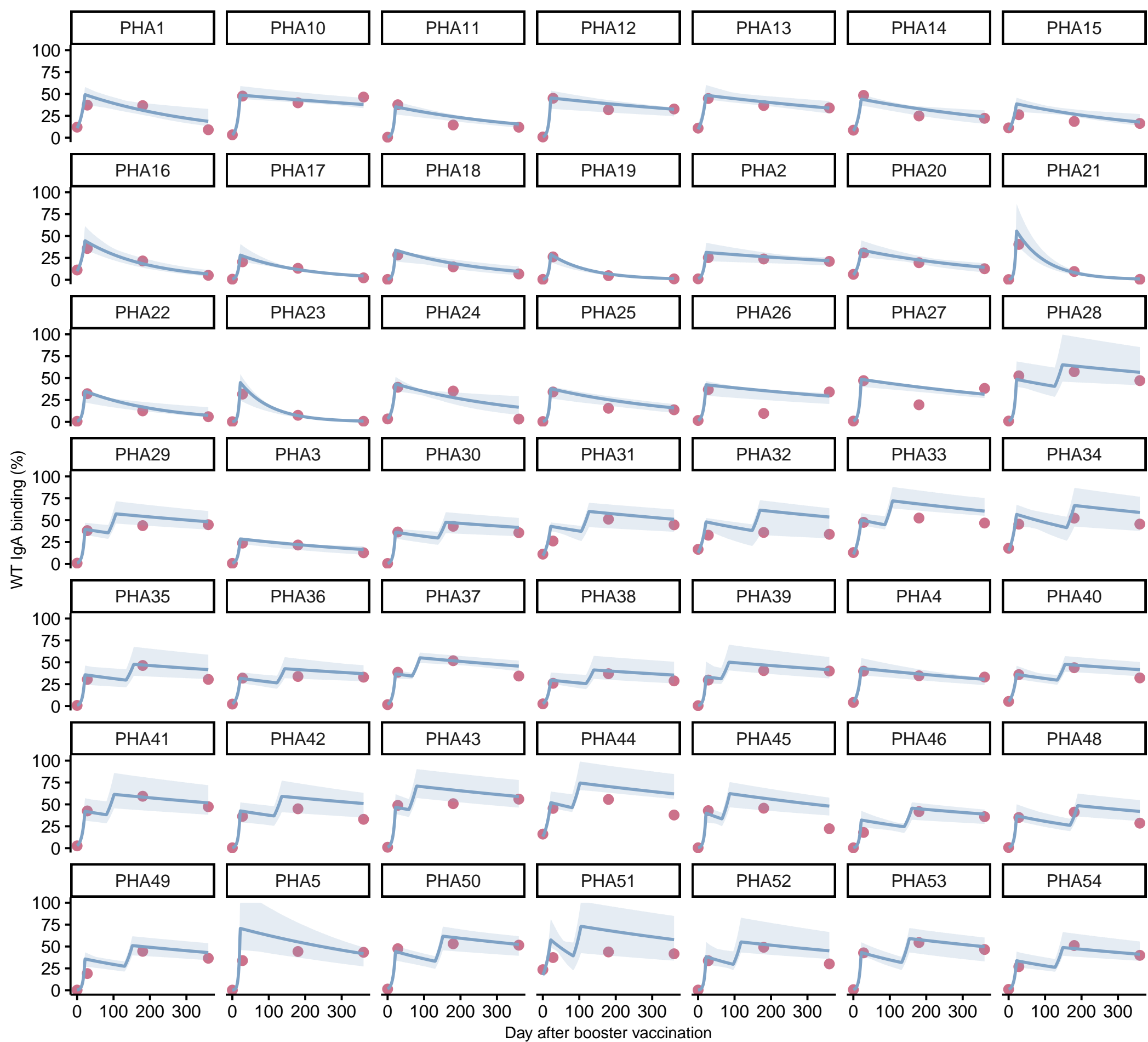

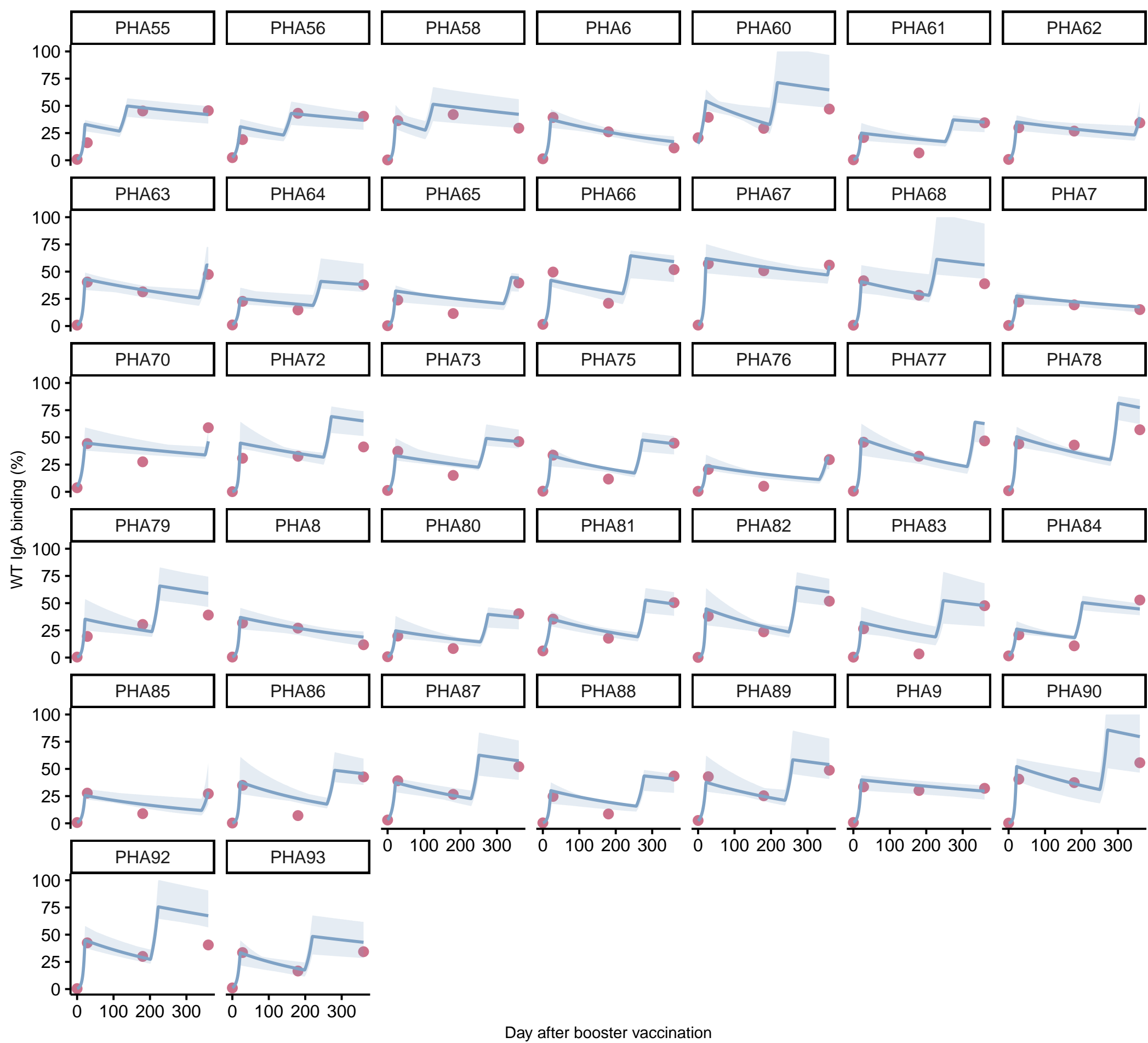

Supplement: Supplementary file 3 — Figure S3: Estimated WT IgA antibody dynamics. [file JMV-97-e70521-s006.pdf]

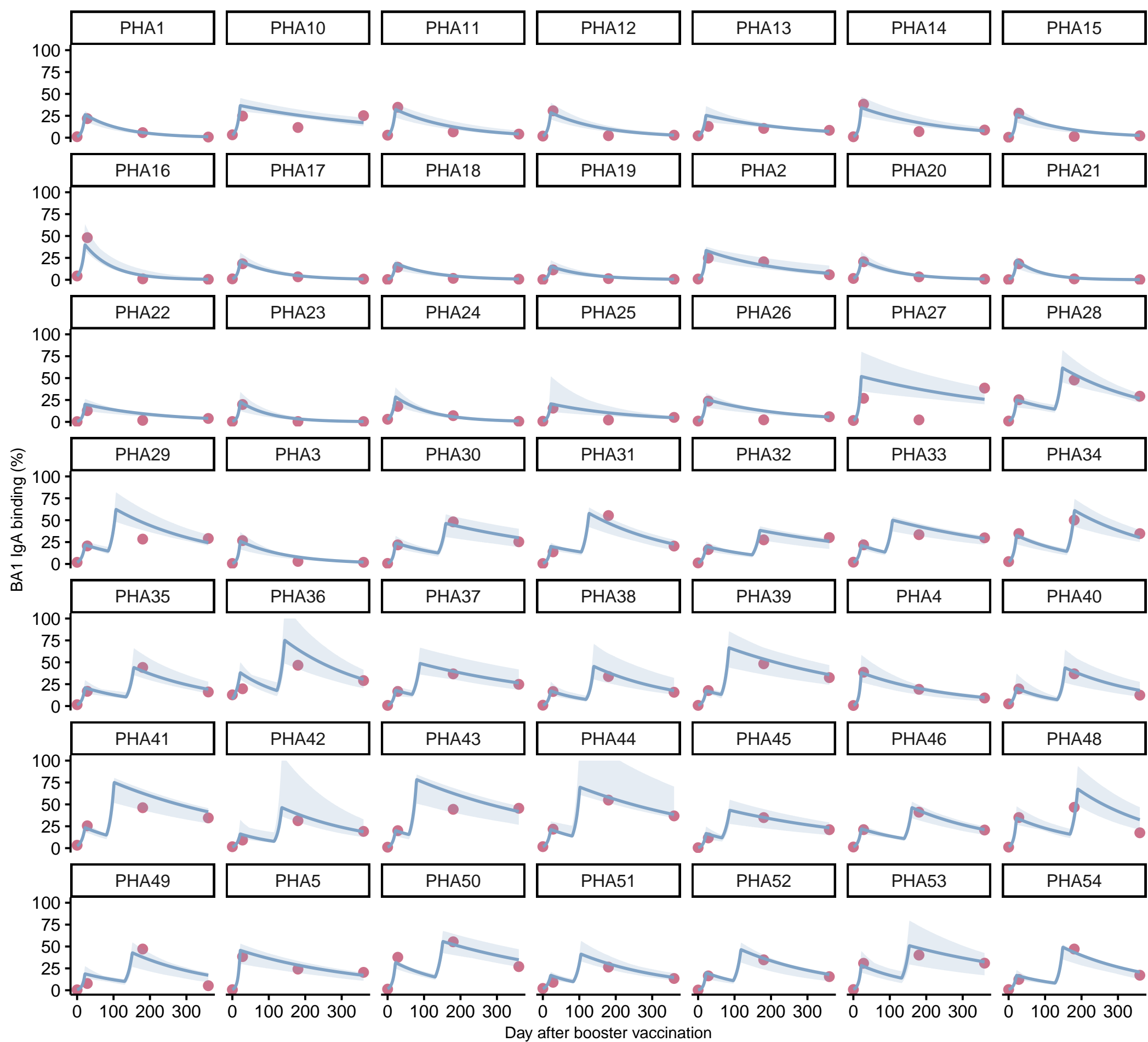

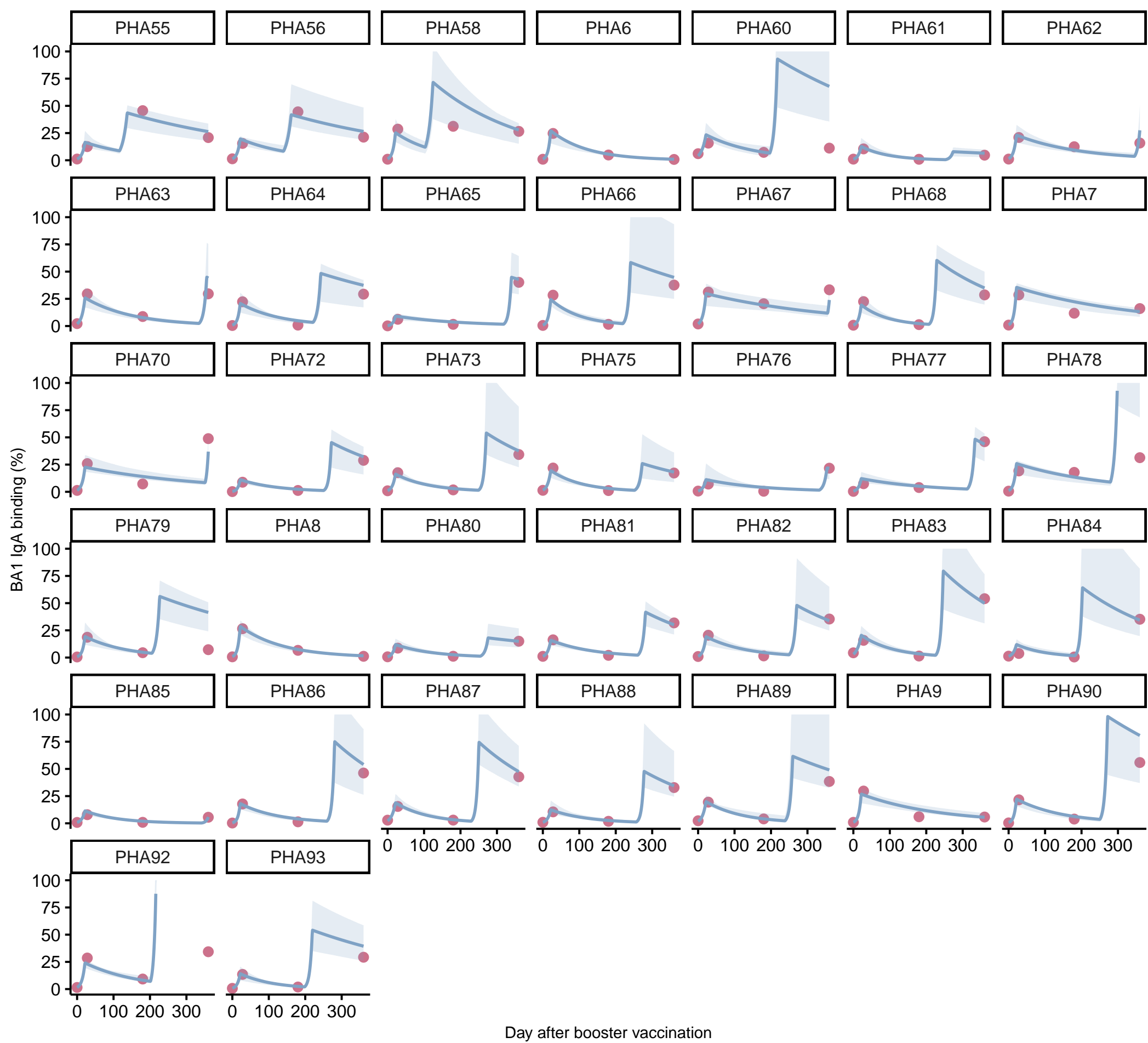

Supplement: Supplementary file 4 — Figure S4: Estimated BA.1 IgA antibody dynamics. [file JMV-97-e70521-s001.pdf]

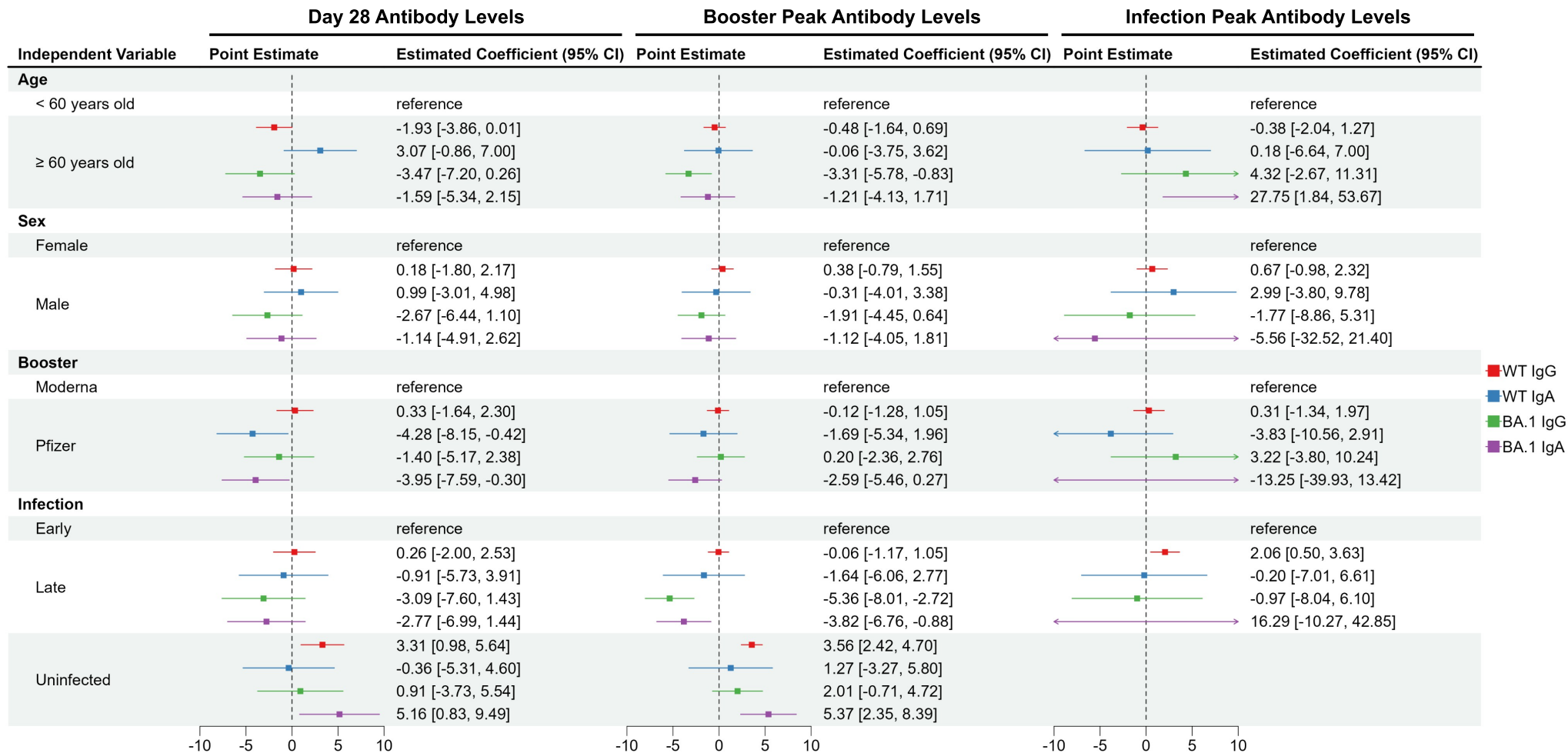

Supplement: Supplementary file 5 — Figure S5: Association between antibody levels and participant characteristics. [file JMV-97-e70521-s005.pdf]
