# Supplementary material for: Modeling Antibody Kinetics Post‐mRNA Booster Vaccination and Protection Durations Against SARS‐CoV‐2 Infection
Source: J Med Virol. 2025 Aug 6;97(8):e70521. doi: 10.1002/jmv.70521 (PMC12327169; doi:10.1002/jmv.70521)
Supplement: Supplementary file 6 — Table S1: Estimated model parameters for the WT IgG antibody model. Table S2: Estimated model parameters for the WT IgA antibody model. Table S3: Estimated model parameters for the BA.1 IgG antibody model. Table S4: Estimated model parameters for the BA.1 IgA antibody model. [file JMV-97-e70521-s002.docx]

**Supplementary Materials**

**Supplementary Figure Legends**

**Figure S1. Estimated WT IgG antibody dynamics.** The blue solid lines are the estimated antibody levels over time drawn using the antibody dynamics model with the best-fit individual parameters for each study participant, while the red dots are the observed antibody levels. The blue shaded areas correspond to 95% prediction intervals.

**Figure S2. Estimated BA.1 IgG antibody dynamics.** The blue solid lines are the estimated antibody levels over time drawn using the antibody dynamics model with the best-fit individual parameters for each study participant, while the red dots are the observed antibody levels. The blue shaded areas correspond to 95% prediction intervals.

**Figure S3. Estimated WT IgA antibody dynamics.** The blue solid lines are the estimated antibody levels over time drawn using the antibody dynamics model with the best-fit individual parameters for each study participant, while the red dots are the observed antibody levels. The blue shaded areas correspond to 95% prediction intervals.

**Figure S4. Estimated BA.1 IgA antibody dynamics.** The blue solid lines are the estimated antibody levels over time drawn using the antibody dynamics model with the best-fit individual parameters for each study participant, while the red dots are the observed antibody levels. The blue shaded areas correspond to 95% prediction intervals.

**Figure S5. Association between antibody levels and participant characteristics.** Results of the univariable linear regression analyses using age group, sex, booster type, and timing of infection for each participant as predictors, with the following outcomes: (i) measured D28 binding antibody levels, (ii) peak binding antibody response after booster vaccination, and (iii) peak binding antibody response after breakthrough infection (for those infected during the follow-up period). Point estimates of the regression coefficients are listed along with 95% confidence intervals in parentheses, with each antibody isotype represented by different colors.

**Supplementary Tables**

| **Table S1. Estimated model parameters for the WT IgG antibody model.** | | | | | | | |
| --- | --- | --- | --- | --- | --- | --- | --- |
| **Parameters^a^** | | | **Initial antibody level** | **Growth rate after booster vaccination** | **Decay rate after booster vaccination** | **Growth rate after breakthrough infection** | **Decay rate after breakthrough infection** |
| **Symbol** | | | $A_{0}$ | $k_{1}$ | $k_{2}$ | $k_{3}$ | $k_{4}$ |
| **Unit** | | | % | day^-1^ | day^-1^ | day^-1^ | day^-1^ |
| **Median of fixed effects^b^** | | | 19.3 (0.82) | 0.04 (2.24×10^-3^) | 3.82×10^-4^ (1.26×10^-4^) | 7.98×10^-3^ (3.83×10^-4^) | 3.45×10^-6^ (2.32×10^-6^) |
| **Covariate effects^c^** | **Age group** | **< 60 years old** | Reference | | | | |
|  |  | **≥ 60 years old** | - | - | - | - | - |
|  | **Sex** | **Female** | Reference | | | | |
|  |  | **Male** | - | - | - | - | - |
|  | **Booster type** | **Moderna** | Reference | | | | |
|  |  | **Pfizer** | - | - | 1.19 (0.30)^*^ | 0.51 (0.10)^*^ | 3.70 (0.64)^*^ |
|  | **Timing of infection^d^** | **Early infection** | Reference | | | | |
|  |  | **Late infection** | - | 0.01 (0.03) | - | 0.52 (0.06)^*^ | - |
|  |  | **Uninfected** | - | 0.08 (0.04)^*^ | - | NA | NA |
| **Standard deviation of random effects^c^** | | | 0.24 | 0.26 | 0.49 | 0.06 | 0.71 |
| **Residual error model parameters^e^** | | | a: 5.71 | | | | |
| ^a^numbers in parentheses are the standard error  ^b^lognormal distributions were assumed  ^c^normal distributions were assumed; covariate effect is interpreted as exp(x) times relative to the reference group  ^d^early infection: infected before 180 days after booster vaccination; late infection: infected between 180-360 days after booster vaccination; uninfected: uninfected over the course of follow-up  ^e^constant error model was assumed; standard deviation of the error: a  ^*^covariates that are statistically significant by the Wald test (*p* < 0.05) | | | | | | | |

| **Table S2. Estimated model parameters for the WT IgA antibody model.** | | | | | | | |
| --- | --- | --- | --- | --- | --- | --- | --- |
| **Parameters^a^** | | | **Initial antibody level** | **Growth rate after booster vaccination** | **Decay rate after booster vaccination** | **Growth rate after breakthrough infection** | **Decay rate after breakthrough infection** |
| **Symbol** | | | $A_{0}$ | $k_{1}$ | $k_{2}$ | $k_{3}$ | $k_{4}$ |
| **Unit** | | | % | day^-1^ | day^-1^ | day^-1^ | day^-1^ |
| **Median of fixed effects^b^** | | | 1.83 (0.33) | 0.13 (8.45×10^-3^) | 1.37×10^-3^  (3.34×10^-4^) | 0.02 (9.36×10^-6^) | 6.63×10^-4^  (1.37×10^-4^) |
| **Covariate effects^c^** | **Age group** | **< 60 years old** | Reference | | | | |
|  |  | **≥ 60 years old** | - | - | - | - | - |
|  | **Sex** | **Female** | Reference | | | | |
|  |  | **Male** | - | - | - | - | - |
|  | **Booster type** | **Moderna** | Reference | | | | |
|  |  | **Pfizer** | -0.84 (0.26)^*^ | 0.26 (0.09)^*^ | 0.69 (0.27)^*^ | 0.27 (5.38×10^-4^)^*^ | 0.21 (0.50) |
|  | **Timing of infection^d^** | **Early infection** | Reference | | | | |
|  |  | **Late infection** | - | - | - | 0.49 (1.41×10^-3^)^*^ | - |
|  |  | **Uninfected** | - | - | - | NA | NA |
| **Standard deviation of random effects^c^** | | | 1.14 | 0.39 | 0.65 | 3.53×10^-4^ | 0.21 |
| **Residual error model parameters^c^** | | | b: 0.27 | | | | |
| ^a^numbers in parentheses are the standard error  ^b^lognormal distributions were assumed  ^c^normal distributions were assumed; covariate effect is interpreted as exp(x) times relative to the reference group  ^d^early infection: infected before 180 days after booster vaccination; late infection: infected between180-360 days after booster vaccination; uninfected: uninfected over the course of follow-up  ^e^proportional error model was assumed; standard deviation of the error: b  ^*^covariates that are statistically significant by the Wald test (*p* < 0.05) | | | | | | | |

| **Table S3. Estimated model parameters for the BA.1 IgG antibody model.** | | | | | | | |
| --- | --- | --- | --- | --- | --- | --- | --- |
| **Parameters^a^** | | | **Initial antibody level** | **Growth rate after booster shot** | **Decay rate after booster shot** | **Growth rate after breakthrough infection** | **Decay rate after breakthrough infection** |
| **Symbol** | | | $A_{0}$ | $k_{1}$ | $k_{2}$ | $k_{3}$ | $k_{4}$ |
| **Unit** | | | % | day^-1^ | day^-1^ | day^-1^ | day^-1^ |
| **Median of fixed effects^b^** | | | 3.38 (0.30) | 0.11 (4.69×10^-3^) | 1.58×10^-3^  (2.95×10^-4^) | 0.01 (1.88×10^-5^) | 1.54×10^-5^  (1.66×10^-5^) |
| **Covariate effects^c^** | **Age group** | **< 60 years old** | Reference | | | | |
|  |  | **≥ 60 years old** | - | - | - | 0.30 (1.21×10^-3^)^*^ | - |
|  | **Sex** | **Female** | Reference | | | | |
|  |  | **Male** | - | - | - | -0.02 (1.27×10^-3^)^*^ | - |
|  | **Booster type** | **Moderna** | Reference | | | | |
|  |  | **Pfizer** | - | - | 0.69 (0.22)^*^ | 0.48 (1.25×10^-3^)^*^ | - |
|  | **Timing of infection^d^** | **Early infection** | Reference | | | | |
|  |  | **Late infection** | - | -0.04 (0.03) | - | 0.70 (1.22×10^-3^)^*^ | - |
|  |  | **Uninfected** | - | 0.03 (0.03) | - | NA | NA |
| **Standard deviation of random effects^c^** | | | 0.65 | 0.26 | 0.69 | 1.32×10^-3^ | 1.49 |
| **Residual error model parameters^e^** | | | a: 0.29 / b: 0.31 | | | | |
| ^a^numbers in parentheses are the standard error  ^b^lognormal distributions were assumed  ^c^normal distributions were assumed; covariate effect is interpreted as exp(x) times relative to the reference group  ^d^early infection: infected before 180 days after booster vaccination; late infection: infected between180-360 days after booster vaccination; uninfected: uninfected over the course of follow-up  ^e^combined error model was assumed; standard deviation of the error: a + b *f*(t_ij_ , φ_i_)  ^*^covariates that are statistically significant by the Wald test (*p* < 0.05) | | | | | | | |

| **Table S4. Estimated model parameters for the BA.1 IgA antibody model.** | | | | | | | |
| --- | --- | --- | --- | --- | --- | --- | --- |
| **Parameters^a^** | | | **Initial antibody level** | **Growth rate after booster vaccination** | **Decay rate after booster vaccination** | **Growth rate after breakthrough infection** | **Decay rate after breakthrough infection** |
| **Symbol** | | | $A_{0}$ | $k_{1}$ | $k_{2}$ | $k_{3}$ | $k_{4}$ |
| **Unit** | | | % | day^-1^ | day^-1^ | day^-1^ | day^-1^ |
| **Median of fixed effects^b^** | | | 1.07 (0.09) | 0.13 (5.33×10^-3^) | 6.14×10^-3^ (6.39×10^-4^) | 0.07 (9.18×10^-4^) | 4.07×10^-3^ (6.16×10^-4^) |
| **Covariate effects^c^** | **Age group** | **< 60 years old** | Reference | | | | |
|  |  | **≥ 60 years old** | - | - | - | 0.21 (0.01)^*^ | - |
|  | **Sex** | **Female** | Reference | | | | |
|  |  | **Male** | - | - | - | -0.10 (0.01)^*^ | -0.61 (0.18)^*^ |
|  | **Booster type** | **Moderna** | Reference | | | | |
|  |  | **Pfizer** | - | - | 0.20 (0.14) | - | - |
|  | **Timing of infection^d^** | **Early infection** | Reference | | | | |
|  |  | **Late infection** | - | -0.07 (0.04) | - | 0.71 (0.01)^*^ | - |
|  |  | **Uninfected** | - | 0.09 (0.04)^*^ | - | NA | NA |
| **Standard deviation of random effects^c^** | | | 0.67 | 0.21 | 0.51 | 6.95×10^-4^ | 0.19 |
| **Residual error model parameters^e^** | | | b: 0.40 | | | | |
| ^a^numbers in parentheses are the standard error  ^b^lognormal distributions were assumed  ^c^normal distributions were assumed; covariate effect is interpreted as exp(x) times relative to the reference group  ^d^early infection: infected before 180 days after booster vaccination; late infection: infected between 180-360 days after booster vaccination; uninfected: uninfected over the course of follow-up  ^e^proportional error model was assumed; standard deviation of the error: b  ^*^covariates that are statistically significant by the Wald test (*p* < 0.05) | | | | | | | |
